# Increased *BRAF* copy number in lung adenocarcinoma

**DOI:** 10.3892/ol.2014.2719

**Published:** 2014-11-20

**Authors:** HIDEFUMI SASAKI, MASAHIKO MAEKAWA, TSUTOMU TATEMATSU, KATSUHIRO OKUDA, SATORU MORIYAMA, MOTOKI YANO, YOSHITAKA FUJII

**Affiliations:** 1 Department of Oncology, Immunology and Surgery, Nagoya City University Graduate School of Medical Sciences, Nagoya, Aichi 467-8601, Japan; 2 GSP Lab Inc., Kawasaki, Kanagawa 212-0032, Japan

**Keywords:** BRAF, lung cancer, adnocarcinoma, copy number, V600E

## Abstract

Point mutation of the *BRAF* gene is a genetic event that occurs in a subset of lung adenocarcinoma cases. For example, *BRAF* V600E is a driver mutation that can be effectively targeted using selective BRAF and/or MEK inhibitors. The present study hypothesized that an increase in *BRAF* copy number may be correlated with certain clinicopathological features of lung adenocarcinoma in Japanese patients. The *BRAF* gene copy number was analyzed using quantitative polymerase chain reaction amplifications in 29 surgically treated lung adenocarcinoma cases without *EGFR* or *Kras* mutations from Nagoya City University Hospital (Nagoya, Japan). Seven *BRAF*-mutant cases were included. Increased *BRAF* gene copy number was identified in three lung adenocarcinoma patients (10.3%), all of which exhibited the V600E mutation. Using fluorescence *in situ* hybridization with *BRAF*-specific and chromosome 7 centromeric probes, increased copy number status was associated with gene amplification or gain of chromosome 7. Although increased *BRAF* copy number was correlated with *BRAF* V600E mutations, numerical changes in BRAF copy number were rare and mild in lung adenocarcinoma, resulting in no significant difference in pathological tumor status or tumor stage.

## Introduction

Despite recent improvements in its diagnosis, lung cancer remains a significant cause of mortality among malignant diseases due to its high incidence rate, malignant behavior and a lack of major advancements in treatment strategies (1). In Japan in 2011, the majority of respiratory surgeries performed were a result of lung cancer (48.9%) and >33,000 patients underwent surgery for lung cancer (2). The clinical behavior of lung cancer is predominantly associated with its stage; thus, the treatment of lung cancer by surgery is only achieved in cases presenting in an early stage (3).

In addition to epidermal growth factor receptor (*EGFR*) and anaplastic lymphoma kinase gene alternations, genomic studies in lung adenocarcinoma have identified other potential therapeutic targets, including activating mutations in *Kras*, *BRAF*, *HER2* and *PIK3CA*, in frequencies >1% (4–6). *BRAF* mutations in lung adenocarcinoma would be of interest as these mutations may be associated with increased sensitivity to agents directly targeting BRAF or BRAF-mediated downstream signaling pathways (7,8). For example, *BRAF* V600E is a driver mutation that can be effectively targeted with selective BRAF and/or MEK inhibitors (9–11). Previous reports identified *BRAF* mutations in 1–4% of cases of lung adenocarcinoma (12–15), and 40–50% of lung cancer cases have been demonstrated to harbor non-V600E mutations distributed in exons 11 and 15 (12–17). A number of these non-V600E mutations exhibit only intermediate or low kinase activity, and the analysis of preclinical data indicates that non-V600E-mutant BRAF kinases may be resistant to BRAF-targeted therapy (17,18).

Although *BRAF* copy number gain has been investigated in thyroid tumors (19), to the best of our knowledge, the association between *BRAF* gene mutation and copy number gain in Japanese lung adenocarcinoma patients has not previously been reported. In the present study, the possibility that *BRAF* copy number gain represents a novel mechanism for *BRAF* gene mutation is investigated. To determine the *BRAF* copy number status in Japanese lung adenocarcinoma patients, quantitative polymerase chain reaction (qPCR) amplification was performed. The findings were compared with the clinicopathological features of the lung cancer patients and data from fluorescence *in situ* hybridization (FISH) performed using *BRAF*-specific and chromosome 7 centromeric probes. Typically, increases in *BRAF* copy number are moderate; however, in V600E lung adenocarcinomas, *BRAF* copy number increases occur with significant prevalence.

## Patients and methods

### Patients

The study group included 29 lung adenocarcinoma patients who had undergone surgery at the Department of Oncology, Immunology and Surgery, Nagoya City University Hospital (Nagoya, Japan) between 2002 and 2011. All tumor samples were immediately frozen and stored at −80°C until assaying.

The clinical and pathological characteristics of the 29 lung adenocarcinoma patients were as follows: Stage I, 16 cases; stage II, six cases; and stage III, seven cases. The mean age of the patients was 67.5 years (range, 47–84 years). Among the 29 lung adenocarcinoma patients, eight were female and 10 were non-smokers. The samples from these patients had previously been analyzed for *EGFR* or *Kras* gene status (20,21) and were considered to be wild-type. This study was approved by the ethics committee of Nagoya City University (Nagoya, Japan) and written informed consent was obtained from all patients.

### PCR assays for BRAF

Genomic DNA was extracted from the lung cancer tissues using the Wizard^®^ SV Genomic DNA Purification system (Promega Corporation, Madison, WI, USA), according to the manufacturer’s instruction. The DNA concentration was determined using a NanoDrop spectrophotometer (ND-1000, version 3.0; Thermo Fisher Scientific, Wilmington, DE, USA) and adjusted to a concentration of 2.5 ng/ml. *BRAF* copy number was analyzed by performing qPCR assays on a 7500 Real-Time PCR system (Applied Biosystems Life Technologies, Foster City, CA, USA) using a QuantiTect SYBR Green^®^ PCR kit (Qiagen, Valencia, CA, USA), with 5 μl DNA from each tumor sample (20,21). The DNA of each tumor sample was quantified by comparing the target locus (*BRAF*) to the reference long interspersed nucleotide element (*Line-1*), a repetitive element for which the copy number per haploid genome is similar in all healthy and neoplastic human cells (22). The quantification was based on a standard curve previously determined from a serial dilution of healthy human genomic DNA (Roche Diagnostics, Indianapolis, IN, USA) and the relative *BRAF* copy number was normalized to the healthy human genomic DNA (calibrator). Furthermore, the change in *BRAF* gene copy number relative to *Line-1* and the calibrator was determined using the following formula: (T BRAF/T Line-1)/(C BRAF/C Line-1), where T and C represent the quantity present in the tumor DNA and the calibrator, respectively. *BRAF* copy number was determined by assaying *BRAF* for each sample using the following primers: Forward, 5′-TCATAATGCTTGCTCTGATAGGA-3′ and reverse, 5′-GGCCAAAAATTTAATCAGTGGA-3′. In addition, the total DNA content was estimated by assaying *Line-1* elements for each sample using the following primers: Forward, 5′-AAAGCCGCTCAACTACATGG-3′ and reverse, 5′-TGCTTTGAATGCGTCCCAGAG-3′. PCR was performed in triplicate for each primer set and the cycling conditions were as follows: Initial denaturation at 95°C for 15 min followed by 40 cycles at 94°C for 15 sec, 56°C for 30 sec and 72°C for 34 sec.

### BRAF FISH analysis

Unstained 5-μm sections of formalin-fixed and paraffin-embedded tumor tissue were submitted to dual-color FISH analysis using four probe sets. The *BRAF/CEN 7q* probe sets were developed at GSP Research, Inc. (Kawasaki, Japan) and were labeled with Texas Red^®^ (TexRed) and fluorescein isothiocyanate (FITC). The probe sets were as follows: BRAF1 (390 kb; 140.3–140.7 MB) at chromosome 7p12-TexRed; and CEN 7q (820 kb; 64.2–65.1 MB)-FITC at chromosome 7q11.21. The lung adenocarcinoma slides were deparaffinized and pre-incubated with Pretreatment Solution (GSP Research, Inc.) at 95–99°C for 30 min, followed by protease digestion buffer at 37°C for 10–20 min. The slides were subsequently washed and dried. In addition, labeled probe sets (10 μl) were cohybridized at 37°C for 72 h following denaturation at 75°C for 5 min. A stringency wash was conducted at 72°C with 2X saline-sodium citrate/0.3% Nonidet P-40 (Sigma-Aldrich, St. Louis, MO, USA) for 1–2 min and the slides were counterstained with DAPI. The slides were then visualized using the Leica MM AF imaging system (Leica Microsystems, Wetzlar, Germany).

### Statistical analysis

Statistical analyses of unpaired samples were performed using the Mann-Whitney U test, and correlation coefficients were determined by rank correlation using Spearman’s rank correlation analysis and the χ^2^ test. All analyses were performed using StatView software (Abacus Concepts, Inc., Berkeley, CA, USA) and P<0.05 was considered to indicate a statistically significant difference.

## Results

### BRAF gene status in Japanese lung adenocarcinoma patients

The clinicopathological data of the 29 lung cancer patients is indicated in Table I. Using primers sets for *BRAF*, 3/29 patients were identified to express >3 copies of the *BRAF* gene. *BRAF* gene copy status was not significantly correlated with gender (male, 9.5% vs. female, 12.5%; P>0.9999), tobacco-smoking (non-smoker, 0% vs. smoker, 15.8%; P=0.5320), pathological tumor (pT) status (pT1, 18.2% vs. pT2–4, 5.6%; P=0.5394), tumor stage (stage I vs. stage II–IV, P=0.9999) or age (<65 vs. ≥65, P=0.5320). No non-V600E *BRAF*-mutant cases exhibited an increased BRAF copy number; however, *BRAF* V600E status was correlated with an *BRAF* increased copy number.

### FISH

**Figure 1:**
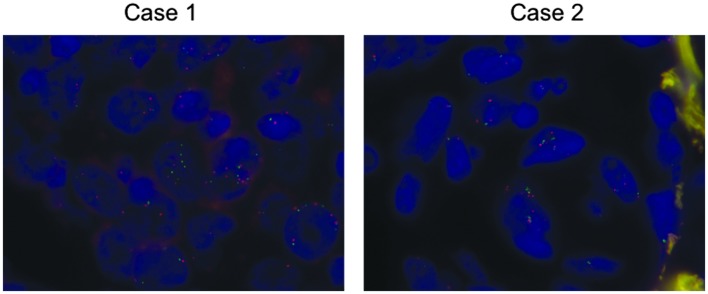
Dual-color fluorescence *in situ* hybridization analysis using the BRAF-specific (red) and chromosome 7 centromeric (green) probes, demonstrating the tumor cells exhibiting amplification (magnification, ×1,000).

The screening of seven *BRAF*-mutant tumors by FISH using a *BRAF*-specific probe revealed two cases (28.6%) with BRAF gene amplification (Fig 1). The two cases were V600E mutants and demonstrated an association between the *BRAF* copy number and chromosome 7 centromeric signals, indicating an association between numerical changes of the BRAF locus and whole chromosome 7 amplification. The *BRAF* copy number in the FISH-positive cases (whole chromosome 7 amplification) was three, 4/5 stage I cases were FISH-negative and 1/2 stage II cases were FISH-positive.

## Discussion

In the present study, increased *BRAF* gene copy number was identified in 10.3% of Japanese lung adenocarcinoma patients without *EGFR* or *Kras* mutations. The *BRAF* gene status was correlated with *BRAF* V600E mutation and whole chromosome 7 amplification.

A previous report demonstrated that the clinical outcomes of *BRAF* mutation-positive patients to platinum-based combination chemotherapy resembled those of wild-type lung cancer patients (23). Within the *BRAF*-mutant cohort, patients with V600E mutations exhibited lower response rates to platinum-based chemotherapy and shorter progression-free survival compared with non-V600E mutation patients (23,24). Previous studies have identified that V600E-mutated tumors are frequently associated with a more aggressive histotype (24,25). Furthermore, current second-generation *BRAF* inhibitors, such as vemurafenib and dabrafenib, have potent, selective activity against the V600-mutant BRAF kinases. One study in the literature described a *BRAF* V600E-mutant lung cancer patient responding to vemurafenib (7) and two studies described a response to dabrafenib (8,26).

Polysomy of chromosome 7 has been identified in the majority of solid tumors (27) and it is well-established that clonal numerical changes of chromosome 7 are common in lung cancer (28,29). Comparative genomic hybridization analysis demonstrated that 65% of lung cancer cases exhibit overrepresentation of chromosome 7p (28). This chromosome 7p gain has been associated with lymph node metastasis in lung cancer (29) and a detailed analysis of chromosome 7 identified various regions of alteration (30), including *EGFR*. Although gains of chromosome 7 result in an increase in the copy number of various genes located on this chromosome, data from the present study indicate that *BRAF* may also represent a target for its selection and clonal progression (19). The present study supports this role of BRAF due to the identification of chromosome 7 amplification in the *EGFR*/*Kras* wild-type, *BRAF* V600E-mutant cases screened. In a previous study, no overlap was identified between *BRAF* copy number changes and *RAS* mutations that are known to activate MAPK (19).

The numerical changes in *BRAF* determined in the present study included gains of three copies of the gene, which would be expected to result in its modest overexpression. However, one of the lymph node-positive V600E cases demonstrated increased copy number. Furthermore, one patient with an increased BRAF copy number had experienced cancer recurrence. Thus, *BRAF* copy number gain may serve as a marker of the more aggressive behavior of V600E lung adenocarcinoma (19).

In conclusion, the present study determined *BRAF* amplification in lung cancer for the first time and demonstrated that BRAF copy number gain may be present in *BRAF* V600E cases. *BRAF* copy number gain is rare in lung adenocarcinomas, however, it does occur in the aggressive V600E subtype.

## Figures and Tables

**Table I:** Clinicopathological data of 29 lung cancer patients.

	*BRAF* gene status		
		
Factor	Increased (n=3)	Normal (n=26)	P-value
Mean age, years^a^ (mean±SD)	75.0±7.0	66.7±9.8	0.1670
Age, years [n (%)]			
<65	0 (0.0)	9 (36.6)	0.5320
≥65	3 (100.0)	17 (65.4)	
Gender, n (%)			
Male	2 (66.7)	19 (73.1)	0.9999
Female	1 (33.3)	7 (26.9)	
Tumor stage, n (%)			
I	2 (66.7)	14 (53.8)	0.9999
II–IV	1 (33.3)	12 (46.2)	
Lymph node metastasis, n (%)			
N0	2 (66.7)	17 (65.4)	0.9999
N^+^	1 (33.3)	9 (36.6)	
Smoking status, n (%)			
Never-smoker	0 (0.0)	10 (38.5)	0.5320
Smoker	3 (100.0)	16 (61.5)	
BRAF mutation, n (%)			
V600E	3 (100.0)	2 (7.7)	0.0027
Non-V600E or wild-type	0 (0.0)	24 (92.3)	
Pathological T status, n (%)			
T1	2 (66.7)	9 (34.6)	0.5394
T2–4	1 (33.3)	17 (65.4)	

a Mean age of total patients, 67.5±9.8 years.

SD, standard deviation; T, tumor.
